# Circadian rhythm as a key player in cancer progression as well as a therapeutic target in *HER2*-positive advanced gastric cancer treatment

**DOI:** 10.3389/fonc.2023.1240676

**Published:** 2023-07-12

**Authors:** Shihao Wang, Suliman Khan, Ghulam Nabi, Hong-Yu Li

**Affiliations:** ^1^ Department of Gastroenterology, The People’s Hospital of Changxing County, Huzhou, Zhejiang, China; ^2^ Medical Research Center, The Second Affiliated Hospital of Zhengzhou University, Zhengzhou, China; ^3^ Institute of Nature Conservation, Polish Academy of Sciences, Krakow, Poland

**Keywords:** circadian rhythm, cancer treatment, epidermal growth factor receptor 2, gastric cancer, trastuzumab

## Abstract

Gastric cancer is one of the most common cancer types with less than one year prognosis in metastatic disease, which poses a huge disease burden. One of the key players in poor prognosis is human epidermal growth factor receptor 2 (*HER2*), which also contributes to the pathogenesis of *HER2*-positive advanced gastric cancer. Trastuzumab is used as first-line chemotherapy that targets the expression of *HER2*, however, trastuzumab resistance is an inevitable major problem. To overcome this problem, readjustment of the circadian system may play a crucial role, as dysregulation in the expression of circadian clock genes has been observed in tumors. Therefore, pharmacological modulation of clock components can be considered for better efficacy of trastuzumab. In this review, we discuss the association of circadian clock with cancer progression, development, and treatment. Metformin-based chronotherapy can disrupt BMAL1–CLOCK–PER1–HK2 axis, thereby affecting glycolysis oscillation to overcome trastuzumab resistance in HER2-positive advanced gastric cancer.

## Introduction

Cancer is the second leading cause of death in the world today, killing millions of people every year (1). According to reports, it is generally associated with disrupted circadian rhythms caused by environmental factors (2). Despite preclinical data supporting this relationship, the exact molecular mechanisms that underlie cancer initiation, development or progression, and clock disruption remain unknown (3, 4). The disruption of circadian rhythms has been linked to breast, prostate, and reproductive cancers in several epidemiological and clinical studies (2, 3, 5). Similarly, earlier reports have indicated molecular evidence linking disruptions of the molecular machinery of the circadian system to cancers, including lung cancer, hepatocellular carcinoma, lymphoma, glioma, and *HER2-*positive advanced gastric cancer (3, 6). A master circadian clock, the suprachiasmatic nucleus (SCN), is an endogenous timekeeper that controls many peripheral clocks throughout the body, where the core circadian genes such as Period 1 (*PER1*) and brain and muscle aryl hydrocarbon receptor nuclear translocator-like protein 1 (*BMAL1*) have been observed for their crucial role in tumorigenesis. (6–8). Melatonin from pineal gland is mitigated by altered dark-light cycles, increasing the likelihood of liver, breast, and other malignancies (9, 10). Circadian rhythm perturbations can induce or increase the risk of cancer by altering antitumor or oncogenes such as growth arrest and DNA damage-inducible (*Gadd) 45a*, cellular myelocytomatosis *(C-myc)*, and *p53* encoding genes (10, 11).

Gastric cancer is one of the most serious cancer types and a challenging global health problem. Each year, over one million people worldwide are diagnosed with gastric cancer, and ranked third death causing cancer. Numerous studies have examined the epidemiology and risk factors of gastric cancer, allowing gastroenterologists to make informed decisions regarding prevention, risk stratification, screening, and treatment (12). However, the high death rate indicates that treatment strategies should be further improved by studying more factors associated with therapeutic efficacy. Among gastric cancers, human epidermal growth factor receptor 2 (HER2)-positive advanced gastric cancer is the most serious cancer type, which lacks effective therapeutic options. The *HER2* gene is altered in more than 10% of advanced gastric cancers, and trastuzumab is the only approved drug that can target HER2, thus being used as a first-line standard treatment option for advanced HER2-positive gastric cancer (13–15). Although, this drug has gained huge recognition among patients with HER2-positive gastric cancer, most patients have been reported to develop resistance to trastuzumab-based treatment. Therefore, researchers have focused on developing new strategies to reverse resistance against trastuzumab resistance in patients. In this regard targeting circadian rhythm is one of the most effective options that can be targeted to improve the efficacy and reverse the resistance against trastuzumab (6, 13). In this review, we discuss the association of circadian rhythm with cancer, targeting the circadian system to improve therapeutic efficacy and reversing the resistance against trastuzumab.

## Mammalian circadian clock

**Figure 1:**
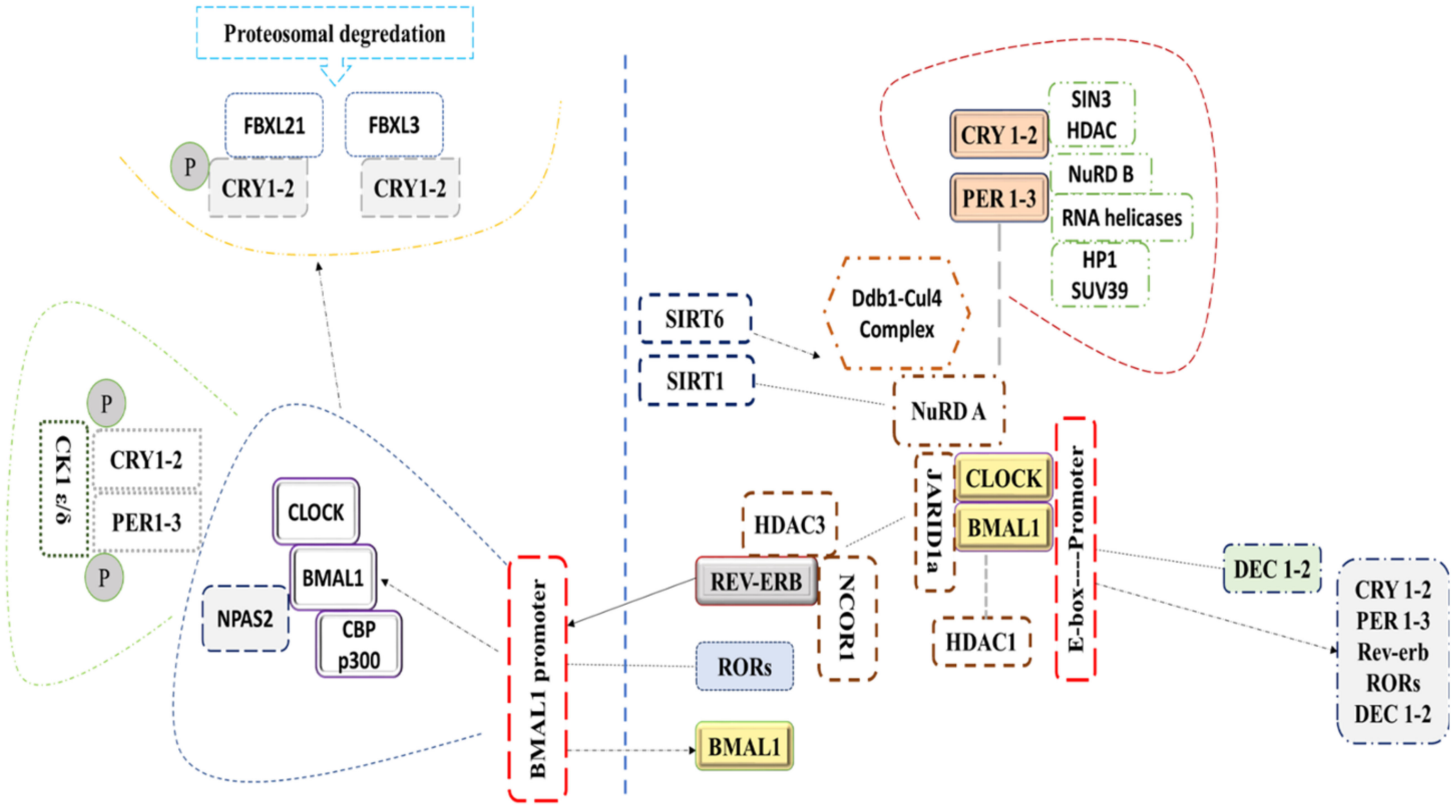
This figure represents the molecular mechanism of the circadian clock in mammals.

Transcription translation feedback loops (TTFLs) of the mammalian molecular clock centering around two transcription factors, BMAL1 and CLOCK (16), which heterodimerize to direct transcription of core circadian clock genes. PER and CRY protein complexes subsequently translocate into the nucleus to inhibit the transcriptional activity of the CLOCK and BMAL1 heterodimer. The complex of CLOCK and BMAL1 are reactivated after repressing the transcription of *PER/CRY* through the degradation of their corresponding proteins (PER and CRY proteins), initiating a new cycle. CRY1 and CRY2 repress transcription in vastly different manners at different phases (**Figure 1**). For instance, the association of CRY2 and PER represses the transcription mediated by E-box in the subjective night by displacing CLOCK and BMAL1 from gene promoters, unlike CRY1, which extends this repression into the early morning by interacting with BMAL1 and CLOCK in a PER-independent fashion (S. 17). In a second feedback loop, CLOCK/BMAL1 complex activates the transcription of *Ror-α* and *Rev-erbα*, that in turn activates and represses *Bmal1’*s transcription, respectively (17). *Rors* and *rev-erba/b* are clock-controlled through E-box elements within their promoters (Bass and Takahashi, 2010; 18). In a third feedback loop, Dec1 and Dec2 proteins displace BMAL1:CLOCK from E-boxes (18, 19) and regulate transactivation of *Per1* (17). Similar to the fly clock, kinases, phosphatases, and ubiquitin ligases modulate the dimerization, subcellular localization, and degradation of PERs and CRYs *via* post-translational modifications (20, 21). Acetylation, phosphorylation, and sumoylation modulate BMAL1/CLOCK transcriptional activity (22, 23).

The endogenous cellular clocks regulate gene transcription (24, 25) in an organ-specific manner (26), where circadian-controlled genes are mostly nonoverlapping in each tissue, which needs to be controlled by cellular physiology across different types of cells. Therefore, the molecular clock is involved in several physiological processes, such as glucose homeostasis and lipogenesis (24, 25). SCN is an autonomous time-keeper, exhibiting circadian rhythm or oscillations in spontaneous firing of action-potential and thereby regulating physiological activities (27–29). In addition to directly targeting other parts of the brain, the SCN neurons produce diffusible molecules that influence behavioral and physiological rhythms (30–32). In order to be effective, biological clocks need to keep accurate time and adjust to environmental signals. Mammalian circadian systems rely on SCN to control peripheral oscillators, and losing SCN results in desynchronized peripheral clocks (30, 33). Nevertheless, tissue-specific gene expression patterns are typically regulated both by “local” and central mechanisms of the circadian system that can interact with peripheral clocks (34). The circadian system and rhythmic metabolic networks are known to be linked by complex feedback loops depending on light conditions. Circadian system-mediated control of metabolism is regulated by the central clocks (SCN) and peripheral clocks located in the pancreas, liver, intestine, skeletal muscle, and adipose tissues. This intimate relationship between metabolism and clocks indicates a role for clocks in regulating gastric functions with the involvement of circadian system regulatory processes, which consist of autonomic innervation of peripheral tissues, temperature, endocrine signaling, and local signals (34, 35).

It is believed that different clocks in various tissues are organized hierarchically, with the master clock being located in the suprachiasmatic nucleus (SCN) of the hypothalamus (29, 30). SCN is both sufficient and essential for generating circadian rhythms (30, 34, 36). The SCN receives direct photic input from photoreceptor cells in the retina termed as the intrinsically photoreceptive retinal ganglion cells (ipRGCs) (29, 34). A photopigment, melanopsin, is present in these ipRGCs, causing them to be intrinsically sensitive to short-wavelength irradiation. An interesting feature of ipRGCs is that they depolarize photoreceptors and utilize phototransduction mechanisms. It is ideal for ipRGCs to function as circadian photoreceptors because they have slow kinetics and a relatively high light threshold. In addition, the sensor must be insensitive to light signals not associated with the solar light cycle and integrate light information over long periods of time. The SCN also receives input from rods and cones, even though ipRGCs are optimal circadian photoreceptors. Interestingly, the ipRGCs mediate these inputs to the SCN from rods and cones. It is emerging that melanopsin-positive ipRGCs modulate a wide range of nonvisual photic responses in mammals (29, 34).

## Circadian clock regulates the progression of cancer

At molecular level, the core circadian clock genes such as *PER1* and *PER2* have been reported to be linked with tumor growth (37). Earlier studies on mice have indicated that altered expression of *Per2* modulates the expression of key tumor suppressors or protooncogenes, including *cMyc, cyclin* A, and mouse double minute 2 homolog or E3 ubiquitin-protein ligase (*Mdm-2*), thereby impairing the apoptosis by p53 gene (35, 38). In human cells, researchers have found that altered expression of *PER1*, *PER2*, and *PER3* can induce the progression of colonic cancer (39, 40) prostate cancer (10) by inhibiting the expression of *P53* and enhances *CMY* expression (38, 41). Moreover, circadian dysregulation suppresses melatonin and related hormones, thereby inducing the progression or occurrence of malignancies (10). One of the important core circadian clock gene, “*BMAL1”* has been found to regulate the molecular processes, thereby preventing or decreasing the risk of cancer progression (42). When circadian rhythms are abnormal, immune function can be downregulated, impairing the immune response against tumors (36, 43). As *BMAL1* regulates inflammation, immune responses, expression of oncogenes and tumor suppressor genes, it may contribute to gastric cancer progression, development, and treatment (42). Similarly, disrupting the clock gene can facilitate tumor cell dissemination into peripheral tissues (44). Nevertheless, cancer progression cannot be attributed to the downregulation of immune function alone. The brain may also trigger signals that may contribute to releasing neurotransmitters and synthesizing mitogenic factors to enhance tumor progression by regulating cancer cell receptors. Furthermore, they suppress hormonal pulsatility, which may promote cell proliferation (44–47).

## Circadian clock as drug target in cancer

Disruption of the cellular pathway of the circadian clock is associated with cancer progression, and the expression levels of core circadian clock genes are largely affected in tumors. The underlying molecular mechanisms through which core clock genes are dysregulated vary in different cancer types based on the nature and location of the tumor. Moreover, the different antitumors or oncogenes may alter the expression of specific circadian clock genes depending on tumor type. Since the circadian clock plays a crucial role in cancer progression, targeting clock genes or the circadian system using effective therapeutic options can improve anticancer treatments (48). Target transcription factors *BMAL1* and *CLOCK* in the human body is challenging because of typical complications in targeting transcription factors mediating protein-DNA or protein-protein interactions. Pharmacological molecules have been developed that can target the activity of the negative regulators of the circadian clock to suppress or promote the transcriptional activity of *BMAL1* and *CLOCK*. Depending on the type of cancer, these clock genes may play an oncogenic or tumor-suppressive role. Therefore, it is necessary to know the exact function of *BMAL1* and *CLOCK* before utilizing clock-targeting compounds to target tumors. In this regard, *REV-ERB* agonists are known clock-targeting small molecules such that GSK4112 or SR6452 synthetic *REV-ERB* agonist can target both REV-ERB isoforms and mimic heme action, which is the physiological ligand for REV-ERBs. It can reset the disrupted circadian rhythms and regulate metabolic pathways but need further improvements to be effective (49). Nonetheless, GSK4112 derivatives “SR9009 and SR9011” have been reported with improved efficacy than GSK4112, and thus be effective against different cancer types with minimum toxicity in animal models (50)(48). However, for convincing antitumor effects, high concentrations of these chemicals were needed, suggesting that further developments or modifications are required to use them as therapeutic options in the clinic (51). The SR9009 may display independent effects of *REV-ERB*, however, the previous report indicated that it retained its activity in mouse hepatocytes under *Nr1d1/2* knockout conditions (52). The loss of *REV-ERBs* causes attenuation of SR9009, suggesting that it may not be ruled out for therapeutic application, however, researchers should further study the exclusive effects of SR9009 on *REV-ERBα/β*. Some of the known small molecule modulators of clock proteins in gastric cancer therapeutics are REV-ERBαagonists “GSK4112 (SR6452) and berberine”, RORα/γ agonist “Nobiletin”, CK1α/δ/ϵ inhibitor “IC261”, and CK2 inhibitor “TBB and CX-4945 (Silmitasertib) (48).

## Treatment of HER-2 positive advanced gastric cancer

The amplification or overexpression of *HER2* or *ERBB2* occurs in approximately 20% of advanced gastric cancers (14). The combination therapy for *HER2*-positive advanced gastric cancer with the anti-*HER2* antibody trastuzumab and chemotherapy has become a first-line standard treatment strategy for patients (53). Interestingly, adding pembrolizumab (anti-programmed death 1 “PD-1” antibody) to chemotherapy cannot improve the treatment in advanced *HER2*-negative gastric cancer, however, it has been found effective in *HER2*-positive gastric adenocarcinoma. Janjiang et al. reported that adding pembrolizumab to trastuzumab and chemotherapy significantly reduced tumour size, improved objective response rate, and induced complete responses in some participants (14). Trastuzumab deruxtecan (DS-8201) consists of anti-HER2 antibody, cytotoxic topoisomerase I inhibitor, and cleavable tetrapeptide-based linker. The efficacy of this drug in patients with *HER2*-positive advanced gastric cancer has been reported, such that therapy with trastuzumab deruxtecan can significantly improve the overall survival compared to typical treatment options (54).

## Circadian rhythm improves treatment of *HER-2*-positive gastric cancer

### Circadian readjustment reverses trastuzumab resistance

**Figure 2:**
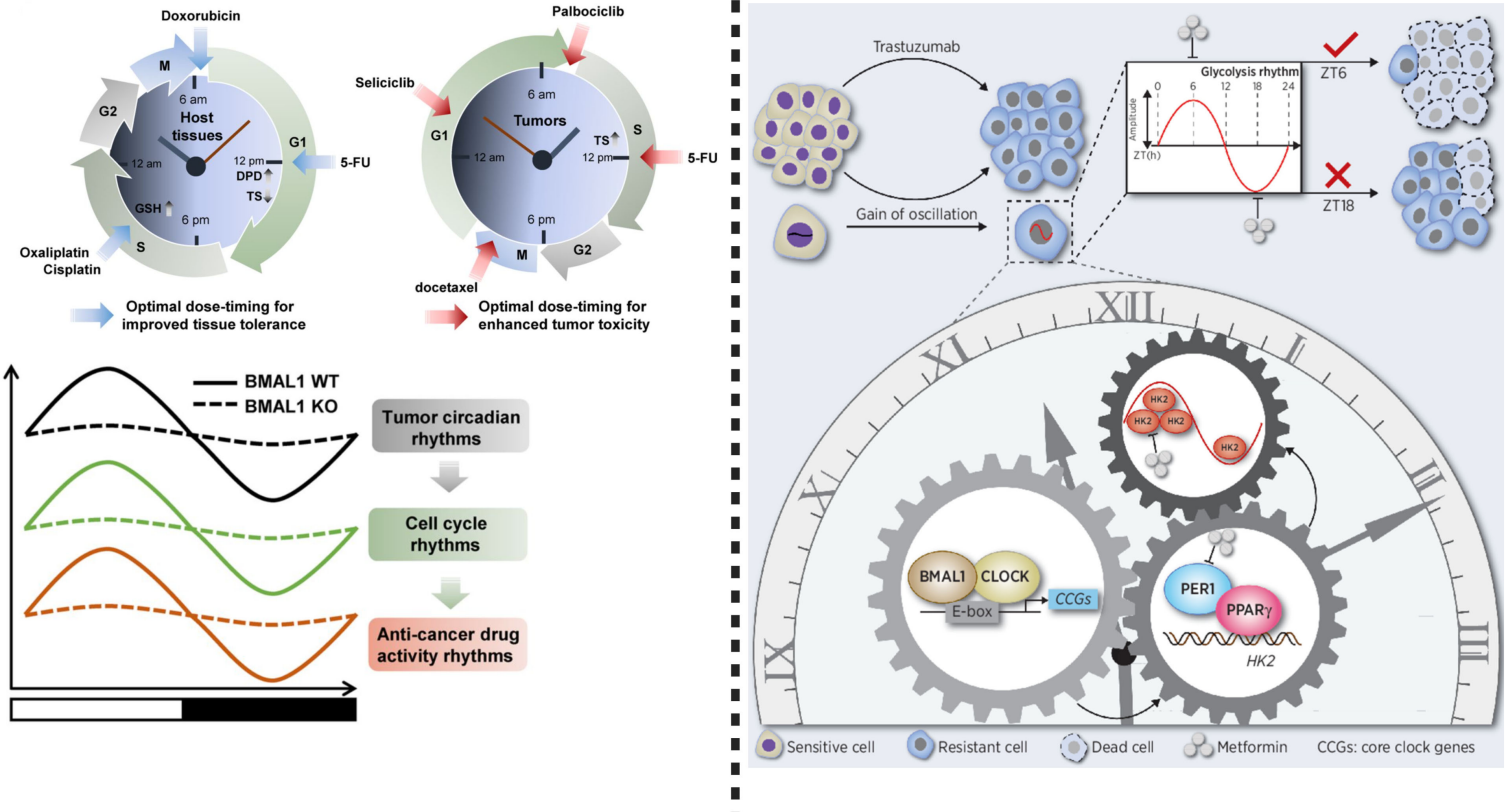
Left: Chronotherapy improves host tolerance and safety against anticancer medicine. To reduce toxicity, dihydropyrimidine dehydrogenase (DPD), 5-FU (an elimination enzyme), and thymidine synthase (TS) play crucial role. Glutathione (GSH), a chronotolerance biomarker is important when oxaliplatin and cisplatin or other platinum based anti-cancer drugs are used. Doxorubicin causes fewer side effects and more therapeutic effects when used in the morning. Circadian rhythms can be targeted by cycle-specific anticancer drugs, while circadian clock function regulates cell cycle rhythms in tumors, which can mediate time-dependent cytotoxicity of antitumor drugs. Importantly, BMAL1 tumor circadian rhythms, cell cycle rhythm and anticancer drug activity rhythm, therefore, targeting BMAL1 in anticancer drug therapy can improve therapeutic response. Right: This figure shows that circadian oscillation of HK2 dependent glycolysis exists in HER2- positive advanced gastric cancer. Chronotherapy using metformin can disrupt rhythm of PER1-HK2, thereby reversing trastuzumab resistance. This modified figure was adopted from Wang et al. (6).

To understand the efficacy and applicability of drugs, it is necessary to understand their mechanism of action and the associated toxicity. Unveiling the mechanism of trastuzumab resistance and its medication under controlled circadian (time-based) conditions may increase sensitivity and reduce toxicity in the case of gastric cancer therapy. The resistance process depends on four central mechanisms including a-*HER2* secondary mutations, b- coexpression of tyrosine kinase receptors, c-bypass signaling activation, and d- factors associated with metabolic processes and tumor microenvironment. Novel agents are being developed to diminish trastuzumab resistance, however, satisfactory outcomes are still limited (6, 13). Nonetheless, in *HER2*-positive advanced gastric cancer, the trastuzumab resistance can be partially reversed by glycolysis reprogramming with the help of oxamate, and glycolysis inhibitor 2-deoxy-D-glucose (2DG). Interestingly, the expression of glycolytic gene “*HKDC1*”, is regulated by the circadian clock (55). Therefore, it is essential to unveil the molecular links between glycolysis rhythm and trastuzumab resistance to utilize chronotherapy targeting metabolic rhythms for overcoming trastuzumab resistance. Wang et al. reported that in trastuzumab-resistant *HER2*-positive gastric cancer, glycolysis activity fluctuates with the circadian rhythm (**Figure 2**), which is mediated by the PER1–HK2 axis through protein–protein interactions between PER1 and PPARg. Here, rhythmic expression of *PER1* or *HK2* may be a candidate biomarker for metformin chronotherapy. This strategy will inhibit glycolysis and degrades PER1 protein to improve trastuzumab efficacy and sensitivity in *HER2-*positive gastric cancer (6).

### Core circadian clock genes regulate oscillation of HK2

To understand the impact of *PER1* regulation, Wang et al. injected *NCI-N87TR/shPER1* (*PER1* knockdown) and *NCI-N87TR/NC* cells into the right and left flank of nude mice, respectively. To determine the impact of PER1 on glycolysis, the levels of glycolysis were measured using F-FDG PET-CT every 4 hours (Total 48 hours) *in vivo*. *HK2* oscillation in TR cells indicates the inter-relationship between the circadian clock and *HK2*. Among the core clock genes (*BMAL1, CLOCK, PER1, PER2, PER3, CRY1, CRY2, NR1D1, NR1D2, DBP, RORα*), the expression rhythm of *PER1* was similar to the oscillation of *HK2*. Core clock genes “*BMAL1*, *CLK*, or *PER1*” can induce the expression and modulate the oscillation of *HK2* such that its expression is downregulated and rhythm is disrupted in response to the silencing of circadian genes. The heterodimer of *BMAL1–CLOCK* binds to E box in the promoter of PER1, thereby activating its transcription to maintain rhythmicity (56)(4, 36). A BMAL1–CLOCK complex modulates PER1 expression and therefore regulates HK2 circadian oscillation, as shown by the reversal of inhibitory effects of siBMAL1 or siCLOCK on HK2 expression following overexpression of PER1. Moreover, silencing or inhibiting the expression of *BMAL1*, *CLK*, and *PER1* not only impairs the activity of *HK2* but also decreases the production of ATP and lactic acid and suppresses glycolysis. It is important to note that enhanced glycolysis activity induces trastuzumab resistance in gastric cancer, and vice versa. A study by Liu et al. (57) concluded that m6A demethylation mediated upregulation of glucose transporter 4 (GLUT4) induces glycolysis and trastuzumab resistance in HER2-targeted therapy. Knockdown or inhibition of GLUT4 decreases glycolysis which in turn can induce sensitivity of cancer cells to trastuzumab (57). Thus, regulating glycolysis is a key factor in developing effective therapeutic strategies against HER2-positive gastric cancer. Overall, these observations indicate that silencing of *BMAL1*, *CLK*, or *PER1* can enhance the response of trastuzumab. On the other hand, *BMAL1–CLOCK–PER1* axis can trigger trastuzumab resistance through the upregulation of *HK2*-dependent glycolysis (6). *HK2* can characterize the glycolysis activity (58), hence *BMAL1–CLOCK–PER1* axis can trigger trastuzumab resistance *via HK2*-mediated glycolysis oscillations in gastric cancer. Metformin, an inhibitor for glycolysis combined with trastuzumab can inhibit cell viability and induce apoptosis. These observations indicate that the combinational effect of metformin and trastuzumab decreases trastuzumab resistance. It is important to note that the overexpression of PER1 successfully reversed these effects. Thus, *PER1* can induce trastuzumab resistance through glycolysis regulation in *HER2*-positive gastric cancer (6).

### *PER1* regulates the rhythmic expression of *HK2*

**Figure 3:**
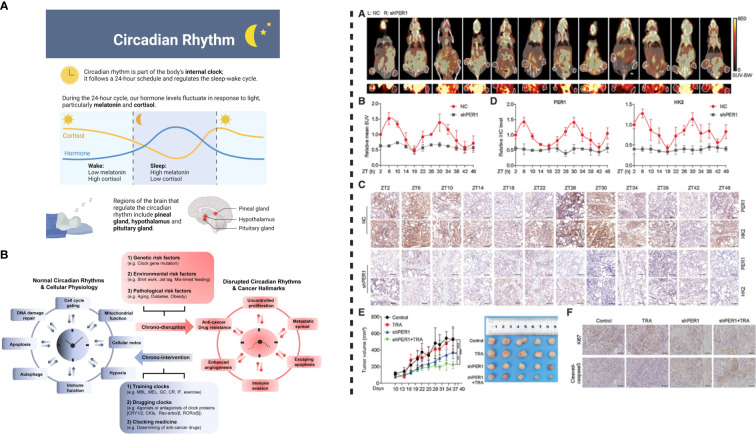
Left: **(A)** Circadian rhythm modulates physiological behavior by regulating hormonal release. **(B)** Circadian system interacts with pathways associated with metabolism and cellular homeostasis. Disrupted circadian clock induces cancer progression and development; normal rhythms can be restored by chronotherapeutic interventions to improve the efficacy anticancer therapeutics/treatments. Right: *PER1* can cause reversal of trastuzumab resistance. **(A)** Injecting transfected NCI-N87TR cells into nude mice and measurement of glycolysis level shown PET-CT images, where white circles detect location of the tumor. **(B)** Standard uptake value (mean values), **(C)** PER1 and HK2 expression indicated by IHC images, **(D)** Levels of PER1 and HK2 detected by quantitative analysis, **(E)** Tumor growth in response to the treatment with trastuzumab and PER1 knockdown, **(F)** Ki67 and cleavage of caspase-3 detected by IHC images. This figure was adopted from Wang et al. (6).

Previous reports have indicated high synchronization in rhythmic expression of *HK2* at the mRNA and protein levels, however, the molecular mechanism underlying *BMAL1–CLOCK–PER1* mediated disruption of *HK2* expression and oscillation is not well understood. Nevertheless, previous studies have concluded that the transcription of circadian clock genes is driven by *BMAL1–CLOCK* heterodimer by binding to the E-boxes located in the promoter, thereby inducing the expression of target genes (56)(4, 36). In a recent study, Wang et al. (6) indicated that in the case of *HER2*-positive gastric carcinoma, *HK2* may not be directly regulated by the binding of *BMAL1–CLOCK* heterodimers to E-boxes. However, it is known that *PER1* can affect transcriptional regulation by interacting with PPARg (59). *PER1* rhythmic oscillation can induce expression of *PPARg*, suggesting that *PER1–PPARg* transcription is under circadian control, which is driven by PER1 protein rather than PPARg. Wang et al. (6) reported that in the case of *PPARg* knockdown, *CD36* was downregulated, however, the knockdown or overexpression of *PER1* did not affect the expression level of PPARg. On the other hand, in repose to the dysregulation of *PER1*, the expression level of *CD36* was altered, indicating that *PPARg* and *PER1* play similar roles. Thus, binding of *PER1* binds to PPARg can affect its activity but not its expression. Still, PPARg plays a crucial role in the transcription of glycolytic isozyme genes *HK2* and *PKM2* (60). In the case of gastric cancer cell lines, *PPARg* silencing can inhibit the expression of *HK2*. PPARg can regulate the transcriptional activity of *HK2* by binding to its promoter regions. Knockdown of *PPARg* by siRNA in *PER1*-overexpressed gastric cancer cells reduces the expression level of *HK2*. Based on the provided information, we can conclude that the binding of PER1 to *PPARg* activates transcription and regulates the rhythmic expression of *HK2* in *HER2*-positive advanced gastric cancer (**Figure 3**).

## Conclusion and perspective

Targeting core circadian components and clock-related proteins can be developed as an anti-cancer strategy or can help explore novel anti-cancer agents. Core circadian components and proteins can be targeted using promising compounds in combination with anticancer agents for a speedy recovery. The development of drugs based on targeting the circadian system may be challenging, such as genetic and pharmacological validation for specific targets in different cancer types may require large-scale investigations. Moreover, unveiling molecular mechanisms of action and determining the tumor-specific therapeutic efficacy of anticancer agents might be needed to address the heterogeneous and conflicting role of clock genes in different cancer types. For future studies, the selection and optimization of most effective molecules will be complicated, as many new compounds are being generated to improve pharmacological performance, and better availability. However, selecting suitable molecule will highly depend on the cancer molecular characteristics associated with clock-targeting agents’ efficacy. Currently, therapeutic effects of metformin combined with trastuzumab are being considered as the most prominent therapeutic option for *HER2*-positive cancer (61), where metformin can restore the sensitivity of trastuzumab under circadian regulation. It is imperative that adjusting the circadian system for metformin can improve its therapeutic effect, and thus this strategy can be employed for other therapeutic strategies in *HER2*-positive advanced gastric cancer. It is known that chemotherapy is also affected by circadian rhythm, such that patients with adenocarcinoma cannot tolerate 5-FU in the early morning. On the other hand, these patients could benefit from the same chemotherapy in the evening (6). Since the therapeutic results for different drugs are not uniform if given simultaneously, a novel approach based on the circadian rhythm of tumor metabolism should be developed to examine drug resistance. In this regard, inhibiting glycolysis at specific time intervals according to the rhythmic expression of *HK2* and glycolysis rhythm reverses trastuzumab resistance and improves its efficacy in *HER2*-positive gastric cancer. Metformin, on the other hand, inhibits *HK2* and induces PER1 degradation, therefore, it can be used as a clock regulator (62). Based on the information provided earlier, we can conclude that the integration of circadian biology into cancer research can be helpful in order to develop novel therapeutic strategies with a unique potential to leverage the interplay between the circadian system and *HER2*-positive advanced gastric cancer. If further studies based on targeting or modulating circadian rhythm as a therapeutic approach are successful. In that case, this approach will significantly improve the anti-tumor treatment efficacy by exploiting distinct targets not only in *HER2*-positive advanced gastric cancer but also other cancer types.

## Author contributions

SW and SK conceived and drafted the manuscript. GN polished and extensively revised the manuscript. H-YL supervised the study. All authors contributed to the article and approved the submitted version. 
